# Supplementary material for: Serum extracellular vesicles profiling is associated with COVID‐19 progression and immune responses
Source: J Extracell Biol. 2022 Apr 20;1(4):e37. doi: 10.1002/jex2.37 (PMC9088353; doi:10.1002/jex2.37)
Supplement: Supplementary file 1 — Table S1: Antibodies and reagents list [file JEX2-1-e37-s003.pdf]

Table S1

Antibodies	Source	Identifier	Concentration	Dilutions
**Serum EVs**				
FITC anti-human CD45 (HI30)	Biolegend	304005	0.5 mg/ml	1/200
PE anti-human CD63 (H5C6)	Biolegend	353003	0.5 mg/ml	1/200
FITC anti-human CD19 (HIB19)	Biolegend	302205	0.5 mg/ml	1/200
PE anti-human CD56 (5.1H11)	Biolegend	981202	100 µg/mL	1/200
FITC anti-human CD31 (WM59)	Biolegend	303103	0.5 mg/ml	1/200
FITC anti-human CD4 (SK3)	Biolegend	980802	50 µg/mL	1/200
PE anti-human CD14 (M5E2)	Biolegend	982508	200 µg/mL	1/200
SARS-CoV-2 Spike S1 Subunit Alexa Fluor® 647-conjugated (Monoclonal Mouse IgG1 Clone # 1035206)	R&D Systems	FAB105403R	100 µg/mL	1/200
PE anti-human CD193/CCR3 (5E8)	Biolegend	310706	0.5 mg/ml	1/200
FITC anti-human CD66b (G10F5)	Biolegend	305104	0.5 mg/ml	1/200
FITC anti-human IgG (M1310G05)	Biolegend	410719	100 µg/mL	1/200
FITC anti-human IgA (IS11-8E10)	Miltenyi	130-114-001	100 µg/mL	1/200
FITC anti-CD38 (HIT2)	Biolegend	303503	100 µg/mL	1/200
**PBMCs**				
Alexa Fluor® 700 anti-human CD45 (HI30)	Biolegend	304023	0.5 mg/ml	1/200
Brilliant Violet 421™ anti-human CD19 (HIB19)	Biolegend	302233	100 µg/mL	1/200
PE/Cyanine5 anti-human CD86 (IT2.2)	Biolegend	305407	100 µg/mL	1/200
PE anti-human IgM (MHM-88)	Biolegend	314507	100 µg/mL	1/200
Goat anti-Human IgA (Polyclonal)	Thermo Fisher	H14001	200 µg/mL	1/200
FITC anti-human CD4 (OKT4)	Biolegend	317407	100 µg/mL	1/200
PE anti-human CD8a (HIT8a)	Biolegend	300907	100 µg/mL	1/200
PE/Cyanine5 anti-human CD69 (FN50)	Biolegend	310907	100 µg/mL	1/200
Brilliant Violet 711™ anti-human IFN-γ (4S.B3)	Biolegend	502539	100 µg/mL	1/200
Brilliant Violet 421™ anti-human TNF-α (MAb11)	Biolegend	502931	100 µg/mL	1/200
