# Supplementary figures and images for: Serum extracellular vesicles profiling is associated with COVID‐19 progression and immune responses
Source: J Extracell Biol. 2022 Apr 20;1(4):e37. doi: 10.1002/jex2.37 (PMC9088353; doi:10.1002/jex2.37)

Figure S1

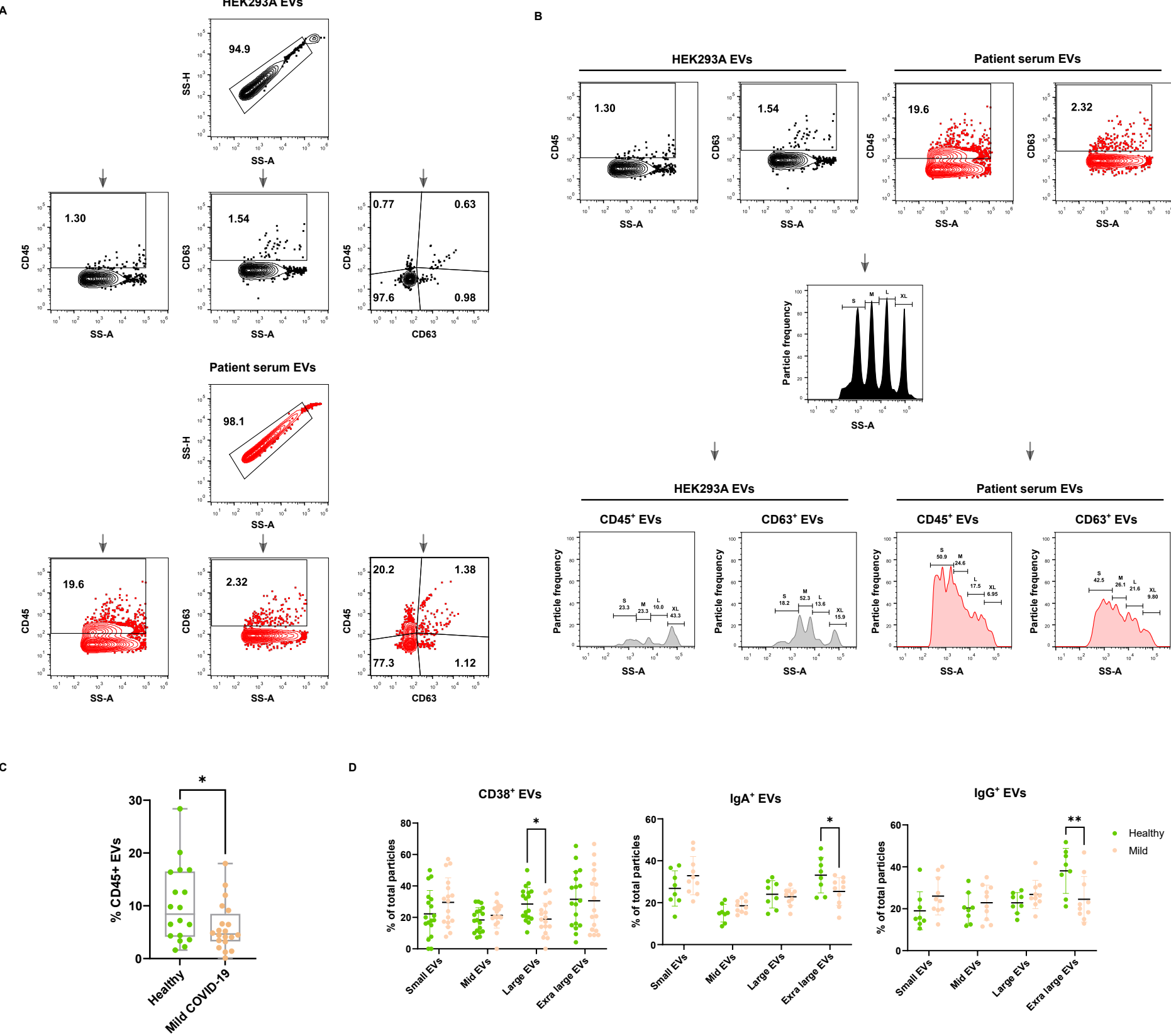

Supplement: Supplementary file 2 — Figure S1. Nanoflow control and experimental setup. (A) Representative nanoflow gating strategies of EVs, HEK293A derived EVs (control) and representative patient derived serum EVs. (B) Representative back gating strategies to determine size distribution of EV subsets using size reference beads with mixture of four modal sizes, 66 nm (small), 91 nm (medium), 113 nm (large), 155 nm (extra‐large). (C) Quantification of CD45+ serum EVs in healthy controls and mild COVID‐19 patients. (D) Quantification of size distribution of CD38+, IgA+, IgG+ serum EVs in healthy controls and mild COVID‐19 patients. [file JEX2-1-e37-s002.pdf]

Figure S3

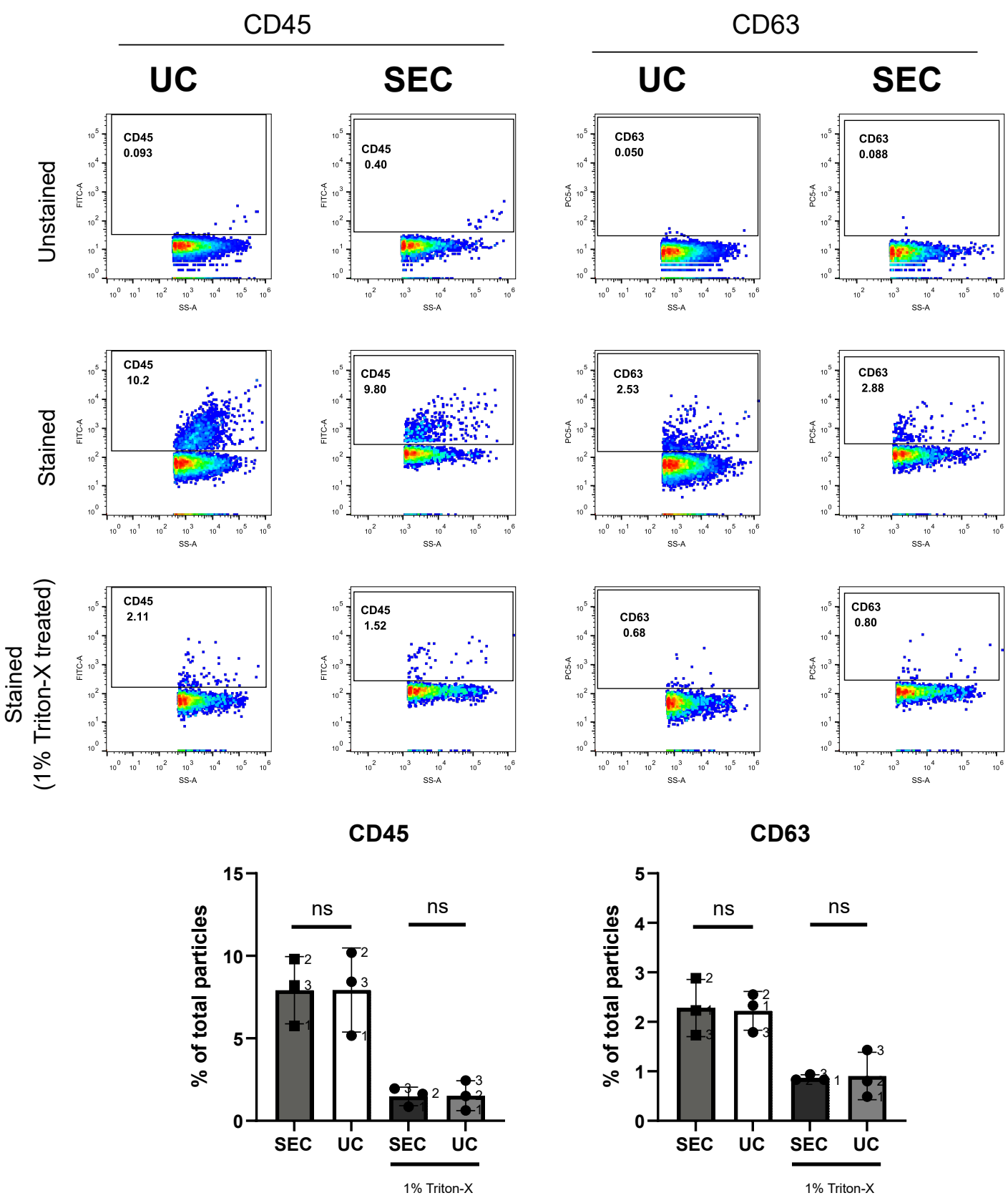

Supplement: Supplementary file 4 — Figure S3. Comparison of EVs markers signals between UC and SEC (Hansa BioMed PURE‐EVs) purification. Representative nanoflow dot plots to assess immunofluorescence intensity (i.e., CD45+ and CD63+ EVs) and purity (1% triton‐X control) of EVs isolated with UC and SEC. Quantification of CD45+ and CD63+ EVs with serum EVs from three independent healthy donors indicated in column graph. [file JEX2-1-e37-s004.pdf]

# Figure S5

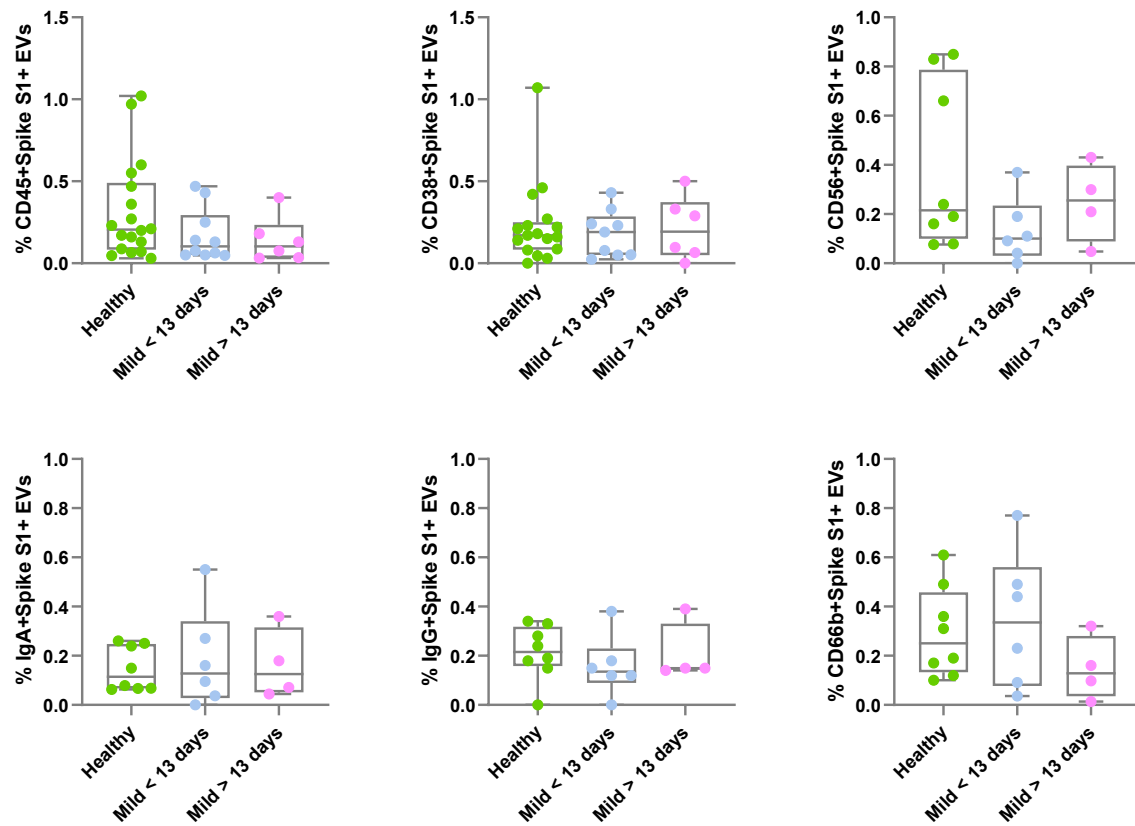

Supplement: Supplementary file 6 — Figure S5. Quantification of Spike S1+CD45+, Spike S1+CD38+, Spike S1+CD56+, Spike S1+IgA+, Spike S1+IgG+, Spike S1+CD66b+ serum EVs in in healthy controls and mild COVID‐19 patients. [file JEX2-1-e37-s006.pdf]

# Figure S6

A

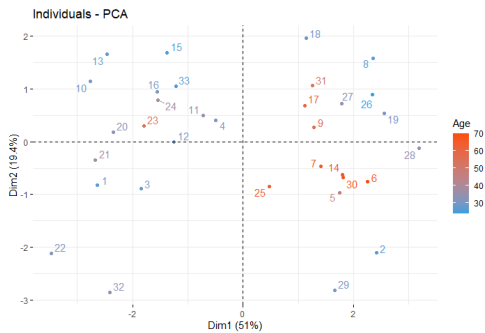

B

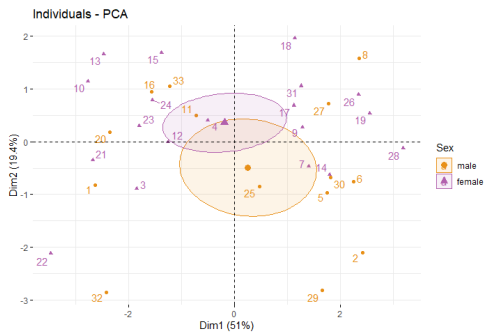

Supplement: Supplementary file 7 — Figure S6. PCA plot clustering of serum EVs samples based on age (A) and sex (B). [file JEX2-1-e37-s001.pdf]
