# Supplementary material for: Serum extracellular vesicles profiling is associated with COVID‐19 progression and immune responses
Source: J Extracell Biol. 2022 Apr 20;1(4):e37. doi: 10.1002/jex2.37 (PMC9088353; doi:10.1002/jex2.37)

Figure S2

A

Nanoflow scatter histograms and dot plots

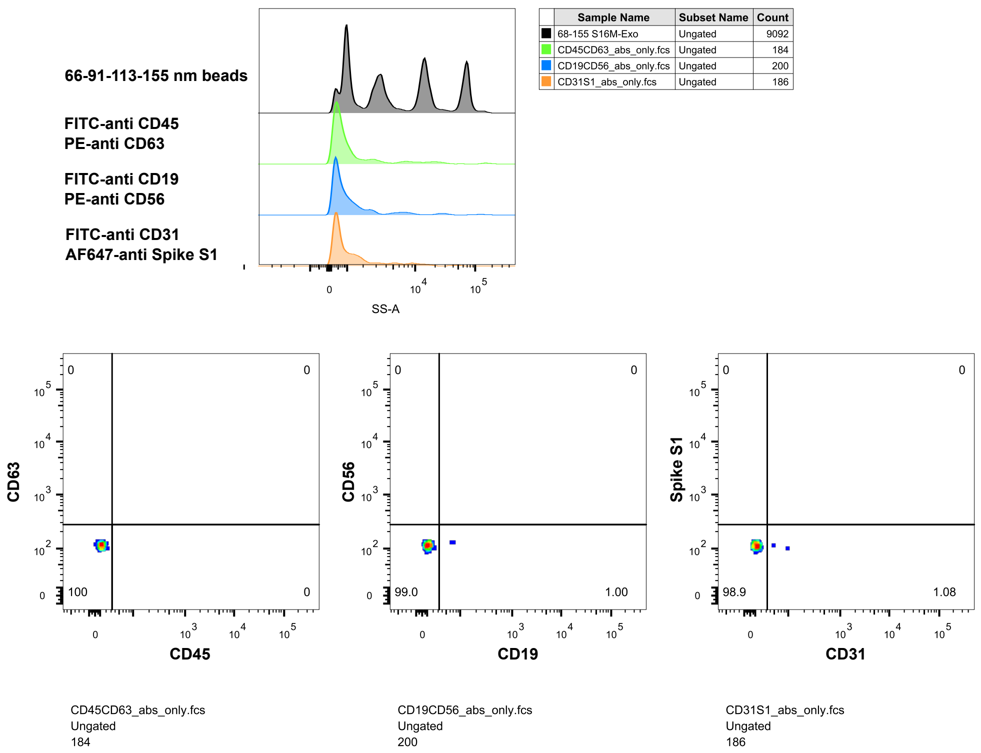

B

Nanoflow scatter histograms and dot plots

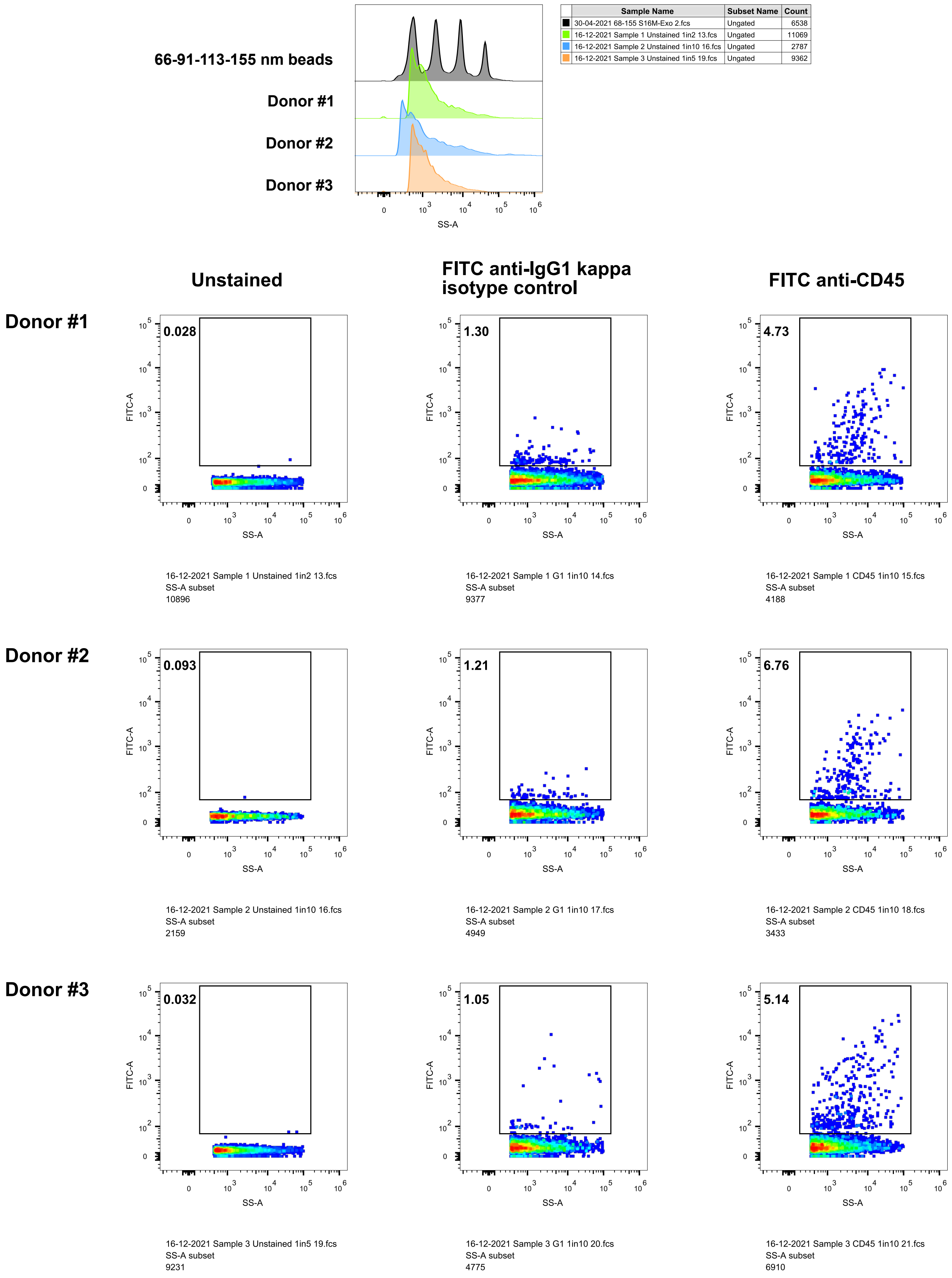

Supplement: Supplementary file 3 — Figure S2. Buffer with reagent controls and antibodies isotype controls. (A) Representative nanoflow scatter histograms and dot plots to confirm effective removal of unbound antibodies or aggregates of antibodies used in the study. (B) Representative nanoflow scatter histograms and dot plots to confirm the specificity of antibodies used in the study. [file JEX2-1-e37-s007.pdf]
