# Supplementary material for: Serum extracellular vesicles profiling is associated with COVID‐19 progression and immune responses
Source: J Extracell Biol. 2022 Apr 20;1(4):e37. doi: 10.1002/jex2.37 (PMC9088353; doi:10.1002/jex2.37)

Figure S4

A

HEK293A co-transfected with GFP and Spike S1 plasmids (0.5 ug)

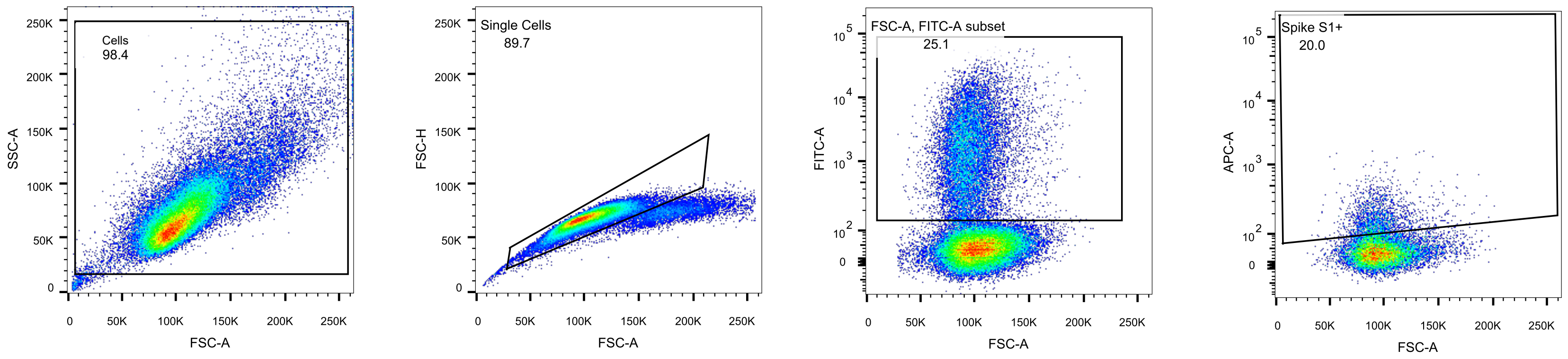

B

Titration of anti-Spike S1 (anti-S1) with recombinant Spike S1 protein (rS1)

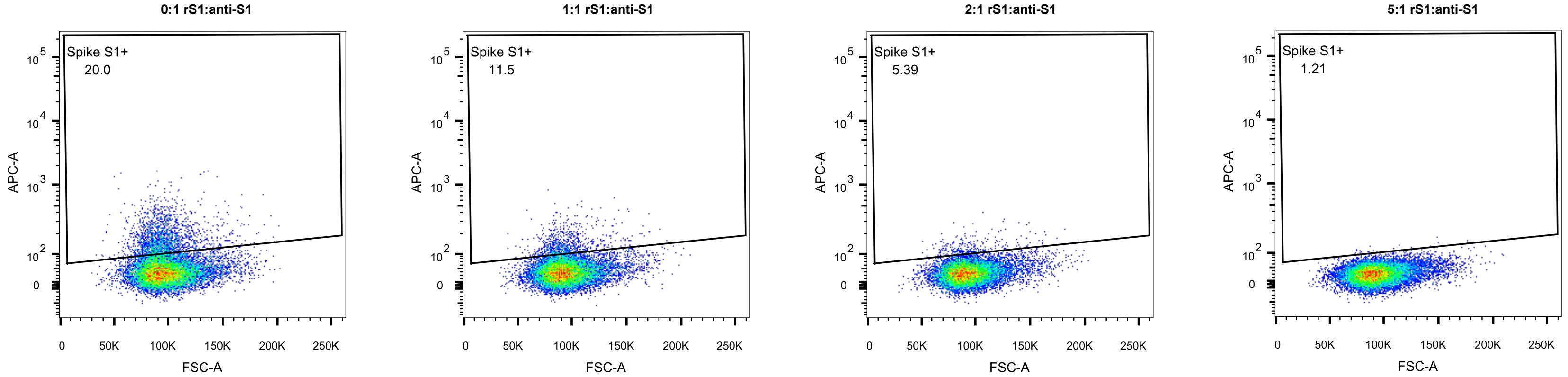

C

S1-HEK293 derived EVs

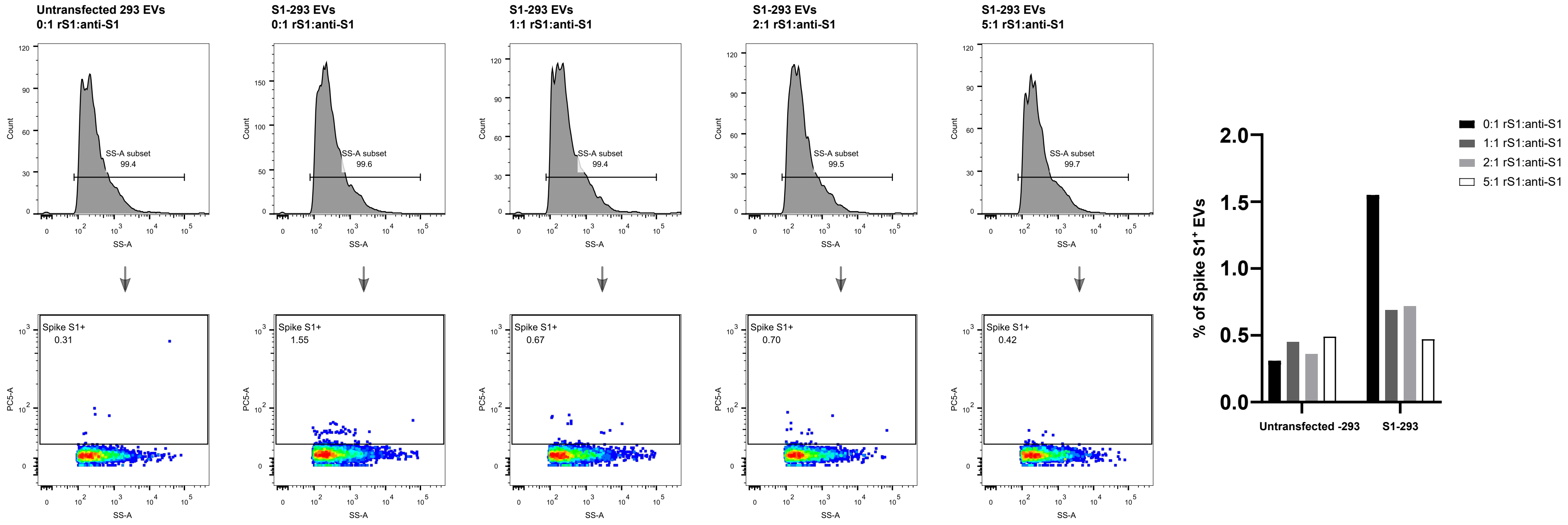

Supplement: Supplementary file 5 — Figure S4. Binding specificity of Sars‐CoV‐2 Spike S1 antibodies. (A) Representative flow gating strategies of HEK293A co‐transfected with GFP and Spike S1 plasmid after 24 h. (B) Competition of anti‐Spike S1 binding in HEK293A in (A) with addition of recombinant Spike S1 proteins in denoted molar ratio. (C) Representative flow gating strategies of EVs derived from HEK293A co‐transfected with GFP and Spike S1 plasmid after 24 h and competition of anti‐Spike S1 binding in EVs with addition of recombinant Spike S1 proteins in denoted molar ratio. [file JEX2-1-e37-s005.pdf]
